# Quantifiable peptide library bridges the gap for proteomics based biomarker discovery and validation on breast cancer

**DOI:** 10.1038/s41598-023-36159-4

**Published:** 2023-06-02

**Authors:** Sung-Soo Kim, HyeonSeok Shin, Kyung-Geun Ahn, Young-Min Park, Min-Chul Kwon, Jae-Min Lim, Eun-Kyung Oh, Yumi Kim, Seung-Man Han, Dong-Young Noh

**Affiliations:** 1 Manufacturing and Technology Division, Bertis Inc., Hungdeok 1-Ro, Giheung-Gu, Yongin-Si, Gyeonggi-Do 16954 Republic of Korea; 2 Bio Convergence Research Institute, Bertis Inc., Heungdeok 1-Ro, Giheung-Gu, Yongin-Si, Gyeonggi-Do 16954 Republic of Korea; 3 Department of Surgery, CHA Gangnam Medical Center, CHA University School of Medicine, 566, Nonhyeon-ro, Gangnam-gu, Seoul, 06135 Republic of Korea; 4 Bertis Inc., 172, Dolma-Ro, Bundang-Gu, Seongnam-Si, Gyeonggi-Do 13605 Republic of Korea; 5 Seoul National University College of Medicine, 103 Daehak-Ro, Seoul, 03080 Republic of Korea

**Keywords:** Breast cancer, Proteomics, Biochemistry, Cancer, Biomarkers

## Abstract

Mass spectrometry (MS) based proteomics is widely used for biomarker discovery. However, often, most biomarker candidates from discovery are discarded during the validation processes. Such discrepancies between biomarker discovery and validation are caused by several factors, mainly due to the differences in analytical methodology and experimental conditions. Here, we generated a peptide library which allows discovery of biomarkers in the equal settings as the validation process, thereby making the transition from discovery to validation more robust and efficient. The peptide library initiated with a list of 3393 proteins detectable in the blood from public databases. For each protein, surrogate peptides favorable for detection in mass spectrometry was selected and synthesized. A total of 4683 synthesized peptides were spiked into neat serum and plasma samples to check their quantifiability in a 10 min liquid chromatography-MS/MS run time. This led to the PepQuant library, which is composed of 852 quantifiable peptides that cover 452 human blood proteins. Using the PepQuant library, we discovered 30 candidate biomarkers for breast cancer. Among the 30 candidates, nine biomarkers, FN1, VWF, PRG4, MMP9, CLU, PRDX6, PPBP, APOC1, and CHL1 were validated. By combining the quantification values of these markers, we generated a machine learning model predicting breast cancer, showing an average area under the curve of 0.9105 for the receiver operating characteristic curve.

## Introduction

Blood proteins are valuable analytes for the diagnosis and prognosis of various diseases^1^. In particular, the application of proteomic platforms to blood proteins has received increasing attention from both academics and clinical industries^2^. With the technological development of mass spectrometry and data analysis methods, MS-based proteomics platforms have gained more depth and quantitative strength to identify and quantify proteins^3^. Accordingly, studies have employed tandem mass tag (TMT)-based methods, label-free quantification methods, and data-independent acquisition (DIA) methods to quantify large number of proteins from complex samples to identify differentially expressed proteins and isoforms as potential candidates for novel biomarkers^3–5^. However, only a small percentage of the candidate biomarkers was identified as effective during the validation phase^1^. This was also observed in the number of biomarkers approved and used clinically. Compared to the over 4,300 plasma proteins identified, only about 100 biomarkers have been approved or cleared by the FDA, despite many discovery studies^2,6,7^. The discrepancy between the discovery and validation phases may be due to differences in the sample size, type, and number, preparation protocol, and equipment^1,8^. Among the processes between the discovery and validation phases, sample size, type, and number can be better controlled at the experimental design stage. However, the differences in preparation methods for different equipment cannot be solved by experimental design. For a typical discovery process, a non-targeted shot-gun proteomic approach using high-resolution mass spectrometry with abundant protein depletion, prefractionation, and a long gradient running time (1–3 h) is used to maximize the number of profiled proteins. In contrast, the validation pipeline is based on a targeted approach on neat serum or plasma via liquid chromatography-triple quadrupole tandem MS (LC–MS/MS), which is more focused on quantitative measurement^9^. The differences between the discovery and validation processes increase time and costs for clinically usable biomarker discovery.

To overcome this problem, previous studies suggested using protocols allowing reproducible analysis in different types of equipment, such as nanoflow and microflow LC^9,10^. These studies focused more on generating a suitable biomarker candidate within a typical discovery setup using an untargeted approach. This may shorten the time of the discovery phase; however, it does not reduce the gap between discovery and validation.

To bridge the gap between discovery and validation, we generated a PepQuant library, which enables the discovery of biomarkers in the setting of a validation process. To construct this library, a list of peptides was first generated and selected from the proteins known to exist or is secreted to blood from public databases and papers. Peptides that are advantageous to be detected by MS/MS were selected, chemically synthesized, and quantified in a 10 min gradient with multiple reaction monitoring (MRM) mode for neat (high-abundant protein undepleted) serum or plasma. This library is thus composed of peptides from the blood protein, that are detectable in a very short gradient time with targeted MRM mode. We next applied the PepQuant library for breast cancer biomarker discovery and validation which resulted in nine final biomarkers. A machine learning (ML) algorithm trained with the identified biomarker candidates discriminated between breast cancer patients and healthy controls with a mean area under the curve (AUC) for the receiver operating characteristic curve (ROC) value of 0.9105.

## Results

### Library generation

**Figure 1:**
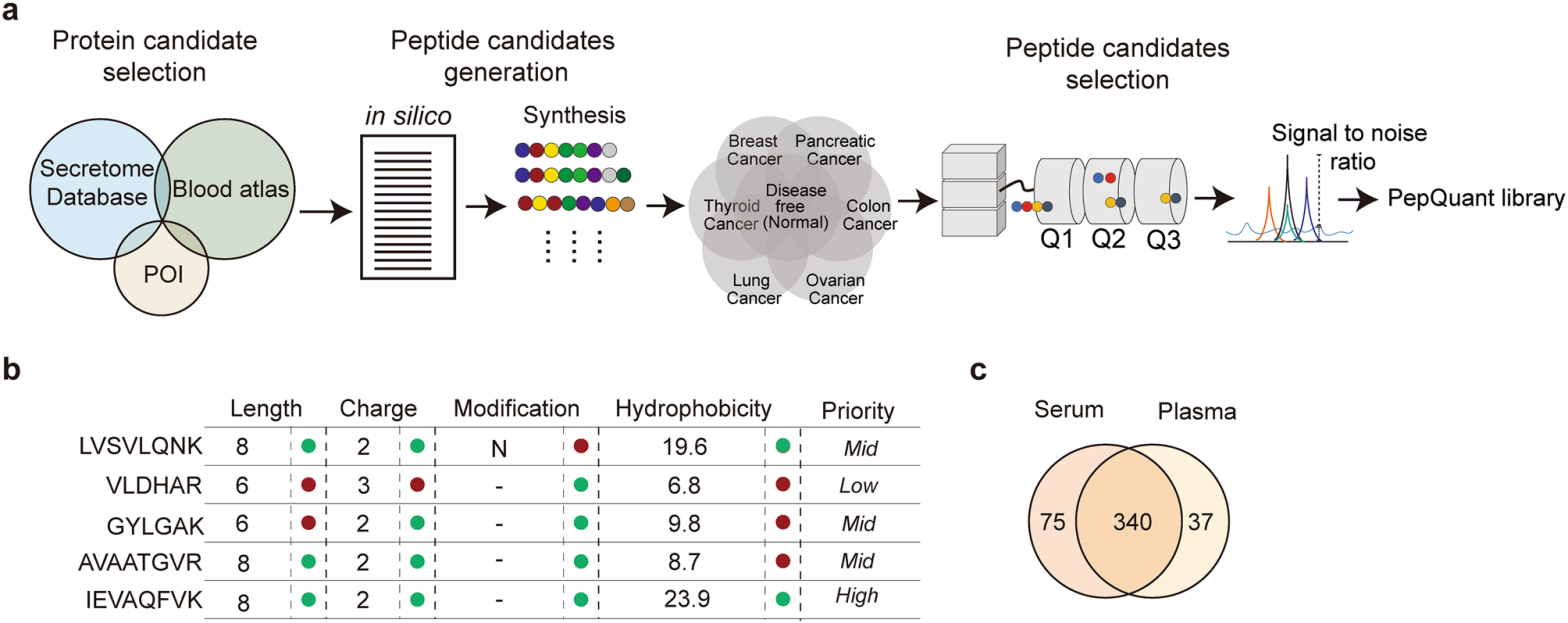
Pep-Quant library generation. (**a**) Schematic diagram showing process of Pep-Quant library generation. (**b**) Schematic diagram showing process of in silico peptide candidate generation from list of proteins in blood. (**c**) A Venn diagram showing number of proteins quantified using Pep-Quant library generation from serum and plasma.

To generate the PepQuant library, we first selected proteins likely to exist in or be secreted into the blood using the human secretome database and Blood Atlas^11,12^. We also added 235 disease-related proteins, resulting in a total of 3393 (Fig. 1a). We created a list of tryptic peptides for each protein from this list, wherein peptide length, hydrophobicity, modifications, and charge were used for selection (Fig. 1b). The selection criteria identified peptides more likely to be detectable in the blood under the harsh condition of a short gradient time and in neat condition, that is serum or plasma used without depletion of the high-abundant proteins. The initial library candidates consisted of 4683 peptides covering 3393 proteins.

To find quantifiable peptides among the 4683 peptide candidates, we first prepared a mixture of 40 breast, 20 pancreatic, 20 thyroid, 20 ovarian, 18 lung, and 20 colorectal cancer samples, along with 30 disease-free samples collected from different hospitals to increase the blood sample diversity. We next analyzed the MS chromatogram for each peptide candidate, by comparing the retention time (RT) of precursor ion and the top three product y-ion peaks between the standard synthetic peptide and the endogenous peptide in the mixture. Among the 4683 peptides, 852 peptides covering 452 proteins were quantifiable with a signal to noise ratio (SNR) above 3, and 95.60% had an SNR higher than 10 (Supplementary Data 1). We also found that approximately 75.22% of the proteins were quantifiable in both plasma and serum, indicating that the library can be applied to for both serum and plasma (Fig. 1c).

### Library characteristics

**Figure 2:**
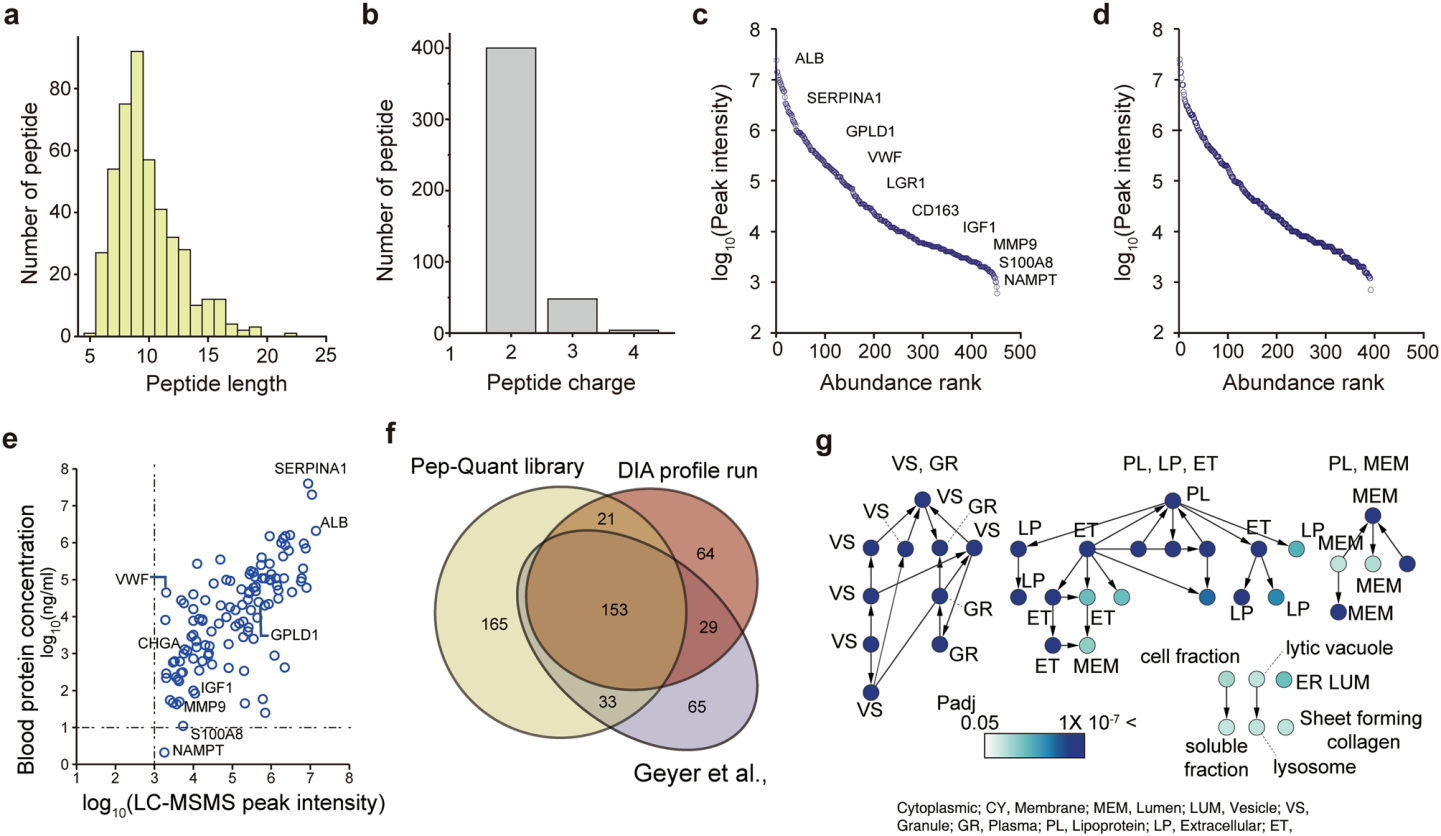
Pep-Quant library characteristics. Bar graph showing distribution of (**a**) peptide length and (**b**) peptide charge. Dot plots showing intensity of peptides in abundance rank for (**c**) serum and (**d**) plasma. (**e**) Dot plot showing comparison between protein concentration from blood atlas, and their reciprocal intensity in MRM mode. (**f**) Venn diagram showing number of proteins commonly found in Pep-Quant library, DIA profile run, and DDA run from Geyer et al.^17^. (**g**) GO functional enrichment network of Pep-Quant library that satisfy a hypergeometric test with false discovery rate correction of P < 0.05. The darker blue color indicates higher enrichment of proteins to function. Only major function or cell components are shown by acronyms; full GO names are shown in Supplementary Fig S1. *CY* cytoplasmic, *MEM* membrane, *Lum* lumen, *VS* vesicle, *GR* granule, *PL* plasma, *LP* lipoprotein, *ET* extracellular.

The PepQuant library was designed to contain peptides 6–16 amino acids long, which are advantageous for detection during LC–MS/MS runs (Fig. 2a,b)^13^. Only 12 library peptides were over 16 or under six amino acids long, as other peptides within the same protein either did not exist or were not detected in the MRM runs. We analyzed the peak intensities in both plasma and serum (Fig. 2c,d) to confirm the dynamic range of the selected peptides, which was approximately 10^3^–10^8^ nm in intensity (Fig. 2c,d). We then compared the intensity values of each peptide with the known concentration of the protein, which did not show a high correlation (Fig. 2e). However, this was expected because the concentration of each protein in the study mixture differed from that in the Blood Atlas. Furthermore, such a difference can occur due to different proteoforms, post-translational modifications, and isoforms^14^.

To verify the coverage of the PepQuant library, we compared the proteins to those identified via the non-targeted approach by the data-independent acquisition (DIA) method using the same concocted samples used to generate the PepQuant library. Among the 850–900 identified proteins, 271 were quantifiable by DIA analysis; among which, 186 proteins were also found in the PepQuant library (Fig. 2f). These data suggest that the PepQuant library covers a similar number of proteins in the human blood, compared to the higher resolution equipment (orbitrap), which uses the DIA method. Next, we compared the proteins in the PepQuant library to those identified by Geyer et al.^15^, where higher-resolution equipment was used to quantify neat blood samples. The proteins in the PepQuant library and profiling were also similar to those found by Geyer et al. despite the difference in sample, methodology, and equipment^15^. These results indicate that the PepQuant library enables the quantification of peptides in the blood with similar level of performance as the higher-resolution equipment.

Next, we investigated the functional enrichment of the PepQuant library using gene ontology (GO). The PepQuant library proteins were enriched for the secretome and extracellular regions, as shown by the clustered networks representing vesicles, granules, lipoproteins, and membranes (Fig. 2g and Supplementary Fig. S1). We did not find enrichment for any single cancer or disease type, which was expected because the proteins in the PepQuant library aim to detect as many quantifiable proteins in the blood as possible without bias to a specific disease.

### PepQuant-library application for breast cancer detection

**Figure 3:**
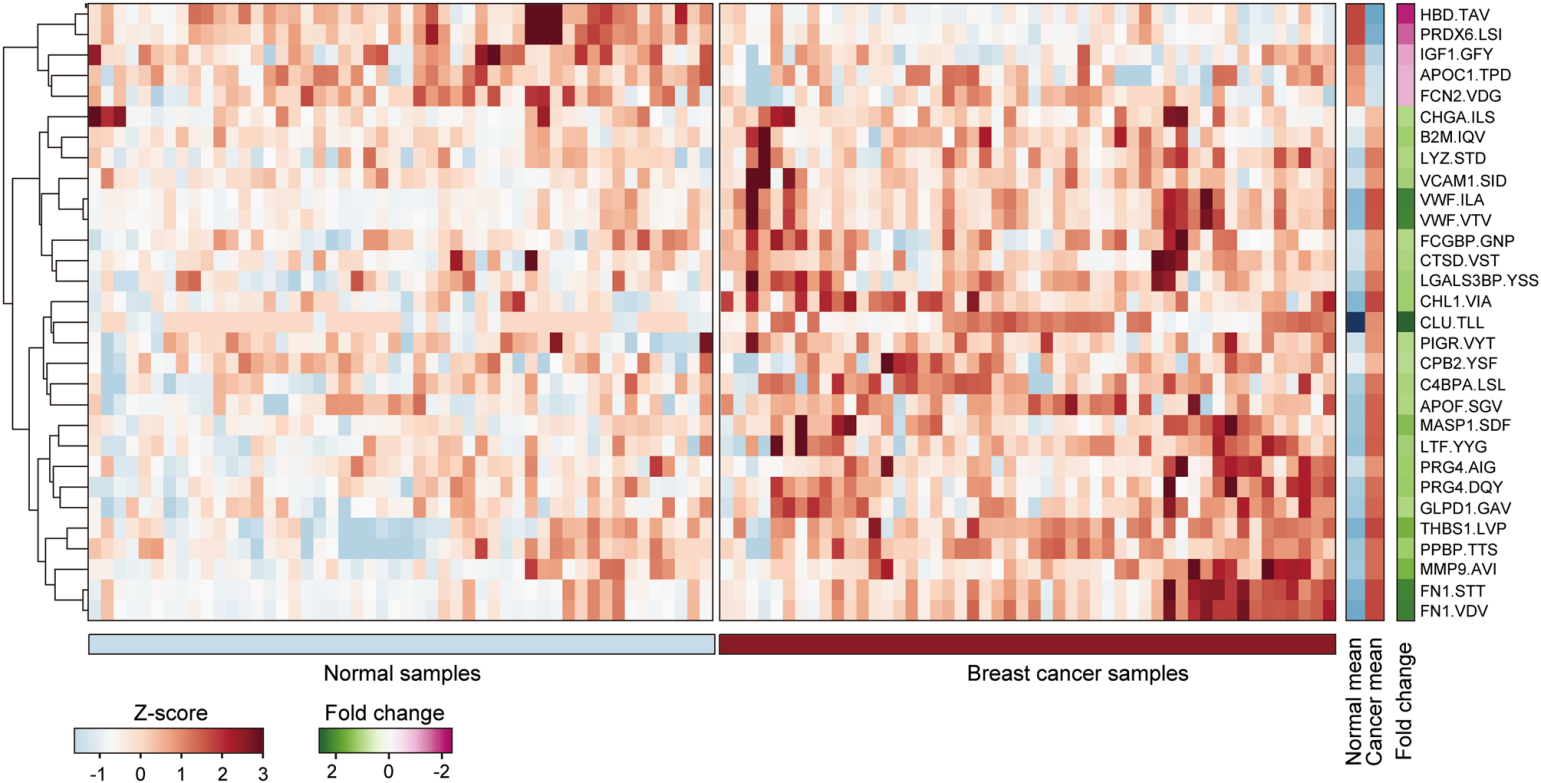
PepQuant library analysis of breast cancer samples. Heatmap showing z-score and fold change values of 30 peptides showing at least 1.2-fold change with a P-value of less than 0.05 for Wilcoxon rank sum test for 50 breast cancer and 50 normal samples. The first three amino acid sequences of each peptide are shown.

To confirm that the PepQuant library enabled rapid biomarker discovery, we analyzed the library against 50 breast cancer and 50 normal serum samples. This resulted in 30 peptides showing at least a 1.20-fold change with a P-value less than 0.05 (Fig. 3 and Supplementary Table S1). We then validated the expression levels of the 30 candidates using LC–MS/MS with a separate and larger scale of another 96 breast cancer and 95 normal samples. Sixteen biomarkers reproduced the fold change cutoffs on a larger scale and thus were subjected to further tests (Supplementary Table S2). To test the usability of the peptides as biomarkers in clinical tests, we proceeded to analytical performance evaluation, testing their precision, stability, and reproducibility under different conditions. Among the 16 peptide candidates, nine showed reproducible quantification results for all tests performed (Supplementary Table S3). The final set of selected biomarkers included FN1, VWF, PRG4, MMP9, CLU, PRDX6, PPBP, CHL1, and APOC1 (Table 1).

**Table 1:** The final validated biomarker-candidates.

Gene	Protein	Peptide sequence
APOC1	Apolipoprotein C1	TPDVSSALDK
CHL1	Neural cell adhesion molecule L1 like	VIAVNEVGR
MMP9	Matrix metalloproteinase-9	AVIDDAFAR
FN1	Fibronectin	STTPDITGYR
VWF	Von Willebrand factor	ILAGPAGDSNVVK
PRDX6	Peroxiredoxin-6	LSILYPATTGR
PPBP	Pro-platelet basic protein	TTSGIHPK
PRG4	Proteoglycan 4	AIGPSQTHTIR
CLU	Clusterin	TLLSNLEEAK

### Breast cancer prediction

We next attempted to generate a ML model for breast cancer prediction using the nine discovered biomarkers. The samples used for training comprised 187 healthy controls and 215 breast cancer samples. A total of 402 samples were used to train several machine learning models; 70% of the pooled samples were used for training and 30% were put aside to be used as test data. To avoid bias, samples were measured in random shuffles with two technical replicates (Supplementary Fig. S2). All algorithms were trained and evaluated five times using the hold-out method (Supplementary Fig. S3). Regardless of the type of ML algorithm, the average AUC value of the prediction exceeded 0.88, higher than the accuracy of molecular-based diagnostic tests of CA15-3 and carcinoembryonic antigen^16^. There was no significant difference in performance between the ML models, indicating that the biomarkers adequately discriminated between the breast cancer and healthy control samples. Among the ML models, the deep learning model showed a slightly higher performance, with a mean AUC of 0.9000 (Supplementary Fig. S3).

**Figure 4:**
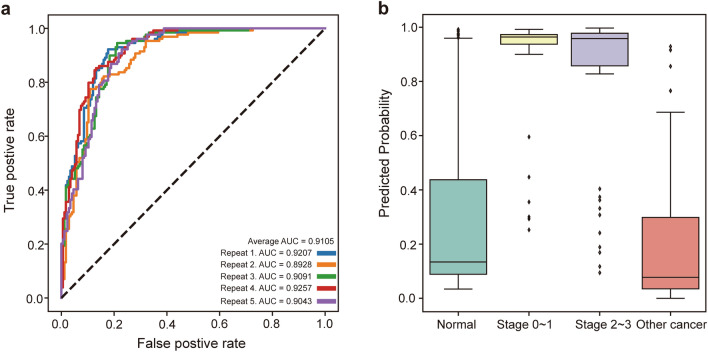
Breast cancer prediction accuracy. (**a**) Area under curve (AUC) receiver operating characteristic (ROC) graph for deep learning. (**b**) Box plot showing predicted probability distribution of breast cancer samples for normal, other cancer and breast cancer for different stages.

We further developed the deep learning model by adding 98 other cancer samples to the original training and test data (Supplementary Table S4). The mean AUC value of the trained model for breast cancer detection was 0.9105, similar to that of the model trained without other cancer data (Fig. 4a). These data suggest that the trained model distinguishes between normal controls and breast cancer samples from data mixed with other cancer samples. To further evaluate the model, we plotted the distribution of the predicted probability of the test data for different stages of breast cancer. The model predicted the early stages of breast cancer in a similar pattern as the later stages (Fig. 4b). Overall, these data indicate that the discovered biomarkers and trained model showed high performance in distinguishing between breast cancer and normal control samples.

## Discussion

**Figure 5:**
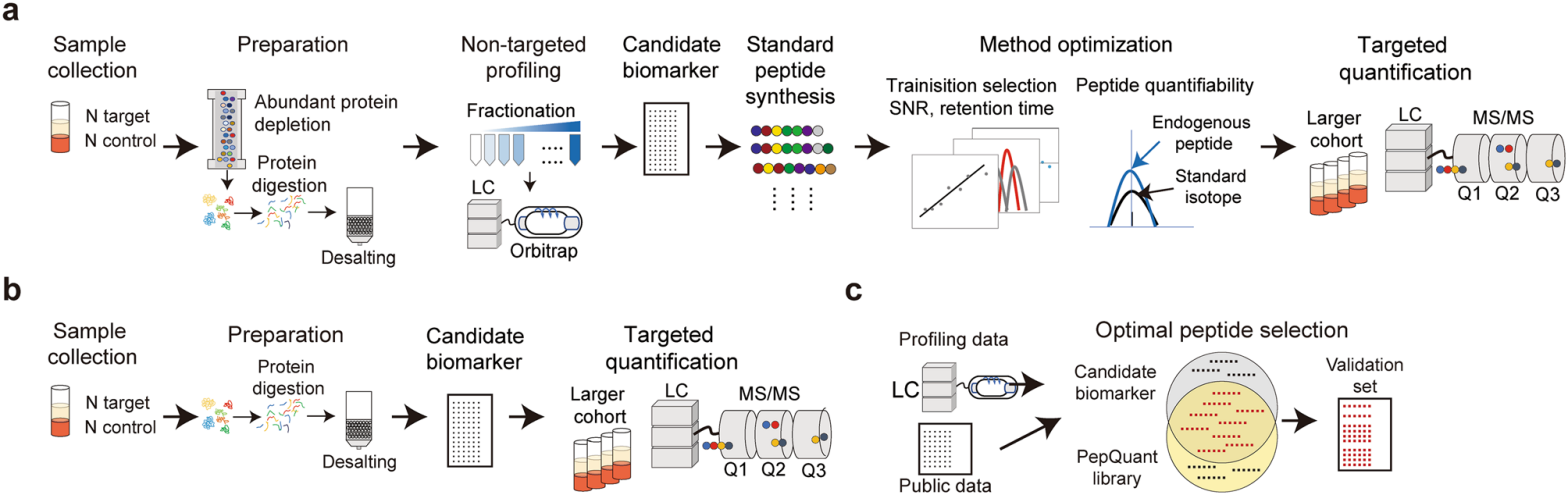
Schematic diagram showing process of discovery and validation of biomarkers for (**a**) typical process and (**b**) using PepQuant library. (**c**) Schematic diagram showing the flow of candidate biomarkers from experiments and public data filtered by PepQuant library.

The PepQuant-library was designed to boost the validation process and increase the number of validated biomarker candidates from discovery. This was achieved by generating a library composed of peptides that have already been confirmed to be quantifiable from the blood in a neat serum or plasma in a 10 min run in MRM mode. The PepQuant library thus allows the process of biomarker discovery in the identical experimental setting as the biomarker validation which significantly reduces the time and cost required to validate each biomarker candidates from discovery. In a typical biomarker discovery and validation study, the number of discovered biomarker candidates may reach up to 50–100. To validate these candidates, first, it would require the synthesis of peptide standards and method optimization for at least 50–100 candidates which may take up to six months (Fig. 5a)^11^. Second, the detectable and quantifiable peptides would need to be quantified again in a larger cohort to confirm reproducibility. However, the PepQuant library allows the skipping of the first step as the method optimization is unrequired and allows to jump directly to the reproducibility confirm step (Fig. 5b). Moreover, the list of peptides in the PepQuant library can benefit future research by providing a list of peptides that are detectable in a validation condition (Fig. 5c).

In this study, nine potential breast cancer biomarkers were discovered using the PepQuant library. All nine biomarker candidates (FN1^17^, VWF^18^, PRG4^19^, APOC1^20^, CHL1^20^, CLU^21^, PRDX6^22^, PPBP^23^, and MMP9^24,25^) are known to be associated to tumor cells and their micro-environmental changes. MMP9 is a metalloproteinase known to degrade extracellular matrix proteins, which is also known to be a step for cancer cell invasion. It has been reported to be upregulated in tumor cells and facilitate EMT (epithelial-mesenchymal transition) or tumor cell migration in breast cancer progression^26,27^. The overexpression of MMP9 was also found in HER2-positive, Triple negative breast cancer and also in metastatic lymph nodes^28^. CLU is a glycoprotein found abundantly in extracellular fluid. It has a chaperone-like properties and plays part in diverse cellular processes such as cell death, inflammation, and tissue remodeling. A study on was conducted on the secretory CLU by overexpression on MCF-7 cell line^29^. The results from the overexpression showed that tumor cells growth rapidly increased and metastasized to the lungs, suggesting significant role of CLU is tumor growth^29^. The role of VWF, PRG4, and PPBP on breast cancer is predicted to be on tumor progression and metastasis. While these three proteins have different functions, all three proteins interact with integrins, which leads to the activation of PI3K/AKT and MAPK signaling pathways that induce cell proliferation^18,19,23,30–32^. Alternatively, PPBP, also known as Chemokine (C-X-C motif) ligand 7 acts on the FAK activation and matrix metalloproteinase promoting migration and invasion^23^. Another study also showed that recombinant PRG4 expression led to the tumor suppression by inhibiting transforming growth factor beta (TGFβ) which led to the decreased hyaluronan (HA)-cell surface cluster of differentiation 44 (CD44)^33^. FN1 interact with different growth factor receptors such as receptor tyrosine kinases and when overexpressed, it leads to unfavorable prognosis for breast cancer^34^. APOC1 and CHL1 have been found in a previous study as biomarkers for a breast cancer in serum which correlates with the discovery and validation of breast cancer biomarkers from PepQuant library^20^.

The nine biomarker candidates for breast cancer are known to be localized in multiple cellular components including extracellular region such as membranes, vesicles and granule and liposome (Supplementary Table S5). They are assumed to secreted to extracellular regions by the canonical secretion pathway through endoplasmic reticulum (ER)-Golgi route. Since the localization and the functional roles of the nine biomarker candidates occur in the extracellular regions, they are detected in serum of normal group as well as in breast cancer group, but differentially expressed. Despite the studied secretion and localization of the biomarker candidates, only a few markers have been previously reported to be as a potential biomarker for breast cancer detectable in neat serum condition. Among them, three breast cancer biomarkers (APOC1, CA1, and CHL1) were found in a previous study and is used as biomarkers for a breast cancer detection algorithm (Mastocheck^®^)^20^. The Mastocheck algorithm performs at a sensitivity of 71.6%, specificity of 85.3%, and AUC of 0.832 in clinical validation studies (normal 122, cancer 183)^35^. In contrast, the ML model developed in this study showed an average sensitivity of 87.9%, specificity of 80.7% and AUC of 0.9105 (Table 2). This result show that the developed ML model with nine biomarkers can be an effective alternative or an assistance blood test for current breast cancer detection system. While effective, the current breast cancer detection heavily relies on the imaging system, which is expensive, carries a risk of radiation exposure and is inaccurate for dense breasts.

**Table 2:** Performance of breast cancer prediction.

Random state	Specificity	Sensitivity	AUC	Accuracy
Random 1st	0.9225	0.8171	0.9207	0.8618
Random 2nd	0.8295	0.8057	0.8928	0.8158
Random 3rd	0.9147	0.7943	0.9091	0.8454
Random 4th	0.8605	0.8229	0.9257	0.8388
Random 5th	0.8682	0.7943	0.9043	0.8257
Average	0.8791	0.8069	0.9105	0.8375

In conclusion, we showed that the PepQuant library can be an effective alternative method for human blood biomarker discovery without high resolution mass spectrometry. By allowing the discovery into a validation set-up where a targeted triple-quadrupole machines is used, it provides more efficiency and reproducibility during validation of biomarkers. With further research, the coverage of the PepQuant-library for the blood proteins and peptides can be improved. While the generated PepQuant-library used public databases on blood and secretome for protein selection, this could be further improved by using more MS/MS databases such as the SRM atlas for peptide selection. Different types of protein databases for membrane or cytoplasmic proteins could be used to expand the PepQuant library. Peptides more suitable for validation setup, quantifiable in MRM mode, higher stability and better representative peptide for a protein, will be researched and added to the library. Overall, we plan to expand PepQuant-library continuously, which would be useful for biomarker discovery and validation research.

## Methods

### Peptide candidate generation

For each protein, a list of all the possible tryptic peptides was generated. The tryptic peptides included all those containing either R or K at both ends, except for sequences containing trypsin-cleavage-resistant amino-acid combinations such as C-terminal RR (arginine-arginine), KK (lysine-lysine), RK, KR, KP and RP. From this list, the peptides with characteristics favorable for detection by MS/MS were selected. The characteristics considered were length, oxidation, post-translational modifications, and hydrophobicity. A higher priority was given to peptides with lengths between six and 16 amino acids, which were detected at higher percentages in a typical MS/MS result compared to other lengths. Extremely hydrophilic or hydrophobic peptides were lower priority because of their lower reproducibility in terms of retention time. Peptides containing possible post-translational modifications, such as glycosylation, and unstable amino acids, such as cysteine (C), methionine (M), or N-terminal tryptophan (W), were given lower priority. For each protein, a peptide candidate was selected for synthesis. Those with similar priorities were selected randomly, and for some proteins, peptide candidates with lower priorities were selected because peptides with higher priorities were missing. Multiple peptides have been synthesized for a few proteins of interest. All peptides were synthesized at the Good Manufacturing Practice facility for medical reagents (Bertis Inc., Korea). The initial library of 4683 peptides were unlabeled and the 452 peptides were isotope-labeled at either Lysine-^13^C_6_, ^15^N_2_ or Arginine ^13^C_6_, ^15^N_4_.

### Peptide candidate selection by MS/MS

To identify quantifiable peptide candidates from blood, we spiked the synthetic standard peptides into the serum and plasma samples to a mixture containing 138 blood samples composed of six different cancer types (40 breast, 20 pancreatic, 20 thyroid, 20 ovarian, 18 lung, and 20 colorectal cancer) and 30 healthy blood samples. The endogenous serum/plasma target peptide spectra were compared to those of the synthetic standard peptides (unlabeled) to identify quantifiable peptide from serum/plasma. To identify the target peptide within the sample, the ratio of the top three peaks of the target peptide for standards and samples were compared (Supplementary Fig. S4a,b). Also, the retention time of the target peptide in the standard, sample, standard spiked in the samples were compared (Supplementary Fig. S4c,d). A peptide was deemed quantifiable when the signal to noise ratio (SNR) was higher than three within a 10 min retention time in an LC run.

### Sample collection

A total of 500 serum samples were collected from 12 Korean hospitals for breast cancer detection. Of these, 215 samples were from breast cancer patients, and 187 were from healthy participants. The remaining 98 samples were from cancer patients from Seoul National University Hospital, with four cancer types: ovarian (20), stomach (20), pancreas (20), lung (18), and colon (20). The healthy samples were listed as category 2 (benign) under BI-RADS (Breast Imaging Reporting and Data System). All samples were from patients who had never been diagnosed with another cancer or had not experienced recurrence within five years.

The samples were collected from August 2019 to September 2020 for a prospective multicenter clinical trial registered at the Clinical Research Information Service of Korea, a member of the WHO International Clinical Trials Registry Platform (ICTRP). The identification number is KCT0004847. The number of samples from each hospital was as follows: Seoul National University Hospital (187), Seoul National University Bundang Hospital (14), Dankook University Hospital (27), Chung-Ang University Hospital (26), Hallym University Gangnam Sacred Heart Hospital (13), National Cancer Center (22), Myongji Hospital (25), Hanyang University Hospital (9), The Catholic University of Korea, Seoul, St. Mary’s Hospital (11), Korea University Anam Hospital (14), Korea University Guro Hospital (29), and Gyeongsang National University Hospital (25). Other cancer serum samples were approved by the Institutional Review Board of Seoul National University Hospital (IRB No. H-1911–085-1079) as non-clinical research using the Human Material Repository. Other cancer serum samples were approved by the Institutional Review Board of Seoul National University Hospital (Approval No. H-1911-085-1079) as non-clinical research using the Human Material Repository. Informed consents were obtained from all participants. This study was conducted in accordance with the Declaration of Helsinki.

### Serum and plasma separation

Whole blood was collected by venipuncture with a 23G syringe and transferred to “vacutainer” serum separation tubes and EDTA blood collection tubes (BD, U.S.A., NJ) for serum and plasma, respectively. They were centrifuged at 2100 × *g* for 20 min at 4 °C, and the supernatant layers were transferred to fresh tubes, and stored at − 80 °C. Prior to mass analysis, the frozen samples were thawed completely at 4 °C and vortexed lightly.

### Sample preprocessing (protein digestion) before MRM analysis

Neat serum samples were directly used without any depletion of high abundant proteins. Five µl of the separated sample were added to an 8 M urea solution containing 18 mM dithiothreitol (Sigma-Aldrich, U.S.A., MA) and incubated for 90 min at 35 °C. The sample is cooled to room temperature and Iodoacetamide (Sigma-Aldrich, U.S.A., MA) is added to a concentration 26 mM and incubated at RT for 30 min in the dark. Ammonium bicarbonate (Sigma-Aldrich, U.S.A., MA) was added (final concentration: 100 mM) to dilute the urea concentration to less than 1 M. Five µg of trypsin (sequencing grade, Promega, U.S.A., WI) was added, followed by incubation at 37 °C for 16 h for protein digestion. Trifluoroacetic acid (Thermo Fisher Scientific, U.S.A., MA) was added to the solution to quench the trypsin activity (final concentration: 1%). Samples were cleaned up using C18 cartridges (Sep-pak C18, 100 mg, Waters) following the manufacturer’s instructions. The cleaned-up samples were dried completely and stored at − 80 °C until use. Prior to MS/MS analysis, dried samples were resuspended in 0.1% formic acid.

### MRM mode mass spectrometry analysis

The mass spectrometer used was a Qtrap5500 Plus (Sciex, U.S.A., MA). For LC separation, a C18 reverse phase column was used (0.5 mm × 150 mm, 3.5 μm, Agilent, U.S.A., CA), and analysis was performed on the positive MRM mode. The flow rate was 20 μL/min, the gradient configuration was set at 5–30% for 0–10 min (10 min gradient time). The mass spectrometric parameter Collision Energy (CE) value for each ionized peptide was determined using SKYLINE software (https://skyline.ms/project/home/begin.view). The mass spectra and chromatography analysis were done using Analyst (1.7.2), and the quantification program used was Multiquant (3.0.2).

### LC–MS/MS deep profiling with data independent acquisition method

The digested peptides were analyzed using a Q Exactive Hf-x Orbitrap mass spectrometer coupled with an Ultimate 3000 UPLC (Thermo Fisher Scientific, U.S.A., MA). For the proteome DIA analysis, the run time was set at 130 min, and the UPLC gradient was set as follows (T min/% of solvent B): 0/3, 5/3, 80/20, 105/40, 105.1/80, 115/80, 115.1/3, 130/3. The peptides were ionized through an EASY-spray column (50 cm × 75 μm ID) packed with 2 μm C18 particles at an electric potential of 1.5 kV. The full MS scan range was set to 300–1400 m/z and the resolution was set to 60,000 at m/z 200. The MS2 scan range was set to 300–1400 m/z, with 44 windows of 25 m/z. The automated gain control target value was set as 3.0 × 10^6^ with a maximum ion injection time of 100 ms.

### DIA analysis

To analyze the DIA data, the raw files were first converted to mzML and imported into DIA-NN^36^. The spectral library comprising 12,046 proteins was downloaded from SWATHAtlas (www.swathatlas.org). A library search was performed according to the DIA-NN manual as previously described^36^. Briefly, the precursor and fragment ion m/z range were set as 300–1400, and the precursor charge range was set as 2–6. Only short-term Methionine excision and Cysteine carbamidomethylation were considered for peptide modification. Up to two missed cleavages were allowed, and the precursor false discovery rate was set to 1%. A default parameter of 0.0 was used for the MS1 accuracy and scan window.

### Breast cancer biomarker discovery and validation

To identify breast cancer biomarkers, all peptides comprising the PepQuant library were tested against 50 healthy and 50 breast cancer patient samples randomly selected from the total samples. Peptides with a fold-change difference of at least 1.2 were selected first. The selected candidates were quantified with additional 95 healthy and 96 breast cancer patient samples. Peptides that satisfy to an at least 1.2 fold-change difference between breast cancer and healthy control samples were subjected to analytical performance evaluation.

### Peptide analytical performance evaluation

The analytical performance evaluation of the LC–MS/MS quantification of protein markers is an essential factor for clinical application^37^. The parameters for analytical performance is mainly consisted in linearity, accuracy, selectivity, precision, and sample stability^9^. The linearity was checked by deriving a linear equation for at least six different concentrations of the peptides and calculating the coefficient of determination (R^2^) between the quantified value and the estimated value obtained from the linear equation. The accuracy was obtained by calculating the ratio between the estimated value from the linear equation to the quantified value for each point of concentration. The peptide was considered acceptable when at least five out of six concentration points were within ± 20% of the accuracy value. The intra-day precision and inter-day precision were tested by repeated measurement of the peptides at different sample concentrations in five technical replicates, within one day and several days, respectively. The stability of the sample peptides was also tested after seven days of storage at 80 °C and 4 °C. For all experiments, isotope-labeled synthetic peptides were used as internal standards (IS). The analyte (peptide) to IS ratio was multiplied by the specific amount of IS to determine the analyte concentration (Supplementary Table S3).

### Diagnostic model development environment

A diagnostic algorithm was developed using deep learning, logistic regression, random forest, and a light-gradient boost algorithm. Logistic regression and Random Forest algorithms were trained with default parameters using ‘Scikit learn v. 0.23.2’^38^. For the gradient boosting algorithm, Python modules ‘Lightgbm v. 3.2.1’ were used. All the machine learning models were tested iteratively using the hold-out method, in which five different random states were used to train and evaluate the algorithm. The deep learning algorithm was developed using Torch v. 1.7.1. Unless otherwise mentioned, all the algorithms were developed using Python v. 3.8.13 environment^39^. The deep learning model structure resembled a GrowNet, which was briefly tweaked to fit the current dataset^40^.

## Supplementary Information

Supplementary Information.

Supplementary Information.

Supplementary Information.

## Data Availability

The data generated in this study are available in the Supplementary Data 2 and uploaded in PASSEL (http://www.peptideatlas.org/passel/), Dataset ID PASS04818.
