# Supplementary material for: Quantifiable peptide library bridges the gap for proteomics based biomarker discovery and validation on breast cancer
Source: Sci Rep. 2023 Jun 2;13:8991. doi: 10.1038/s41598-023-36159-4 (PMC10238494; doi:10.1038/s41598-023-36159-4)
Supplement: Supplementary file 3 — Supplementary Information. [file 41598_2023_36159_MOESM3_ESM.docx]

**Quantifiable peptide library bridges the gap for proteomics based biomarker discovery and validation on breast cancer**

**Sung-Soo Kim^1,2,^**^†^**, HyeonSeok Shin^2,^**^†^**, Kyung-Geun Ahn^1^, Young-Min Park^1^, Min-Chul Kwon^1^, Jae-Min Lim^1^, Eun-Kyung Oh^1^, Yumi Kim^3^, Seung-Man Han^4^ and Dong-Young Noh^3,4*^**^*^

^1^ Manufacturing and Technology Division, Bertis Inc., Hungdeok 1-ro, Giheung-gu, Yongin-si, Gyeonggi-do, Republic of Korea
^2^ Bio Convergence Research Institute, Bertis Inc., Heungdeok 1-ro, Giheung-gu, Yongin-si, Gyeonggi-do, Republic of Korea
^3^ Department of Surgery, CHA Gangnam Medical Center, CHA University School of Medicine, Seoul, Republic of Korea

^4^ Bertis Inc., 172, Dolma-ro, Bundang-gu, Seongnam-si, Gyeonggi-do, Republic of Korea

Keywords: peptide library, Breast cancer, Serum biomarkers, Deep-learning, clinical proteomics, discovery, validation.

Financial support: This work was fully supported by Bertis Inc.

^†^ These authors contributed equally to this work.

**^*^** Corresponding author:

Dong-Young Noh; Chief Executive Officer of Bertis Inc; 172, Dolma-ro, Bundang-gu, Seongnam-si, Gyeonggi-do 13605, Republic of Korea; (Email) [dongyoung.noh@bertis.com](mailto:dongyoung.noh@bertis.com) (Tel) +82-10-5221-9688 (Fax) +82-31-693-8594; Professor emeritus of Seoul National University College of Medicine, 103 Daehak-ro, Seoul 03080, Republic of Korea.

**Supplementary figure S1**

**
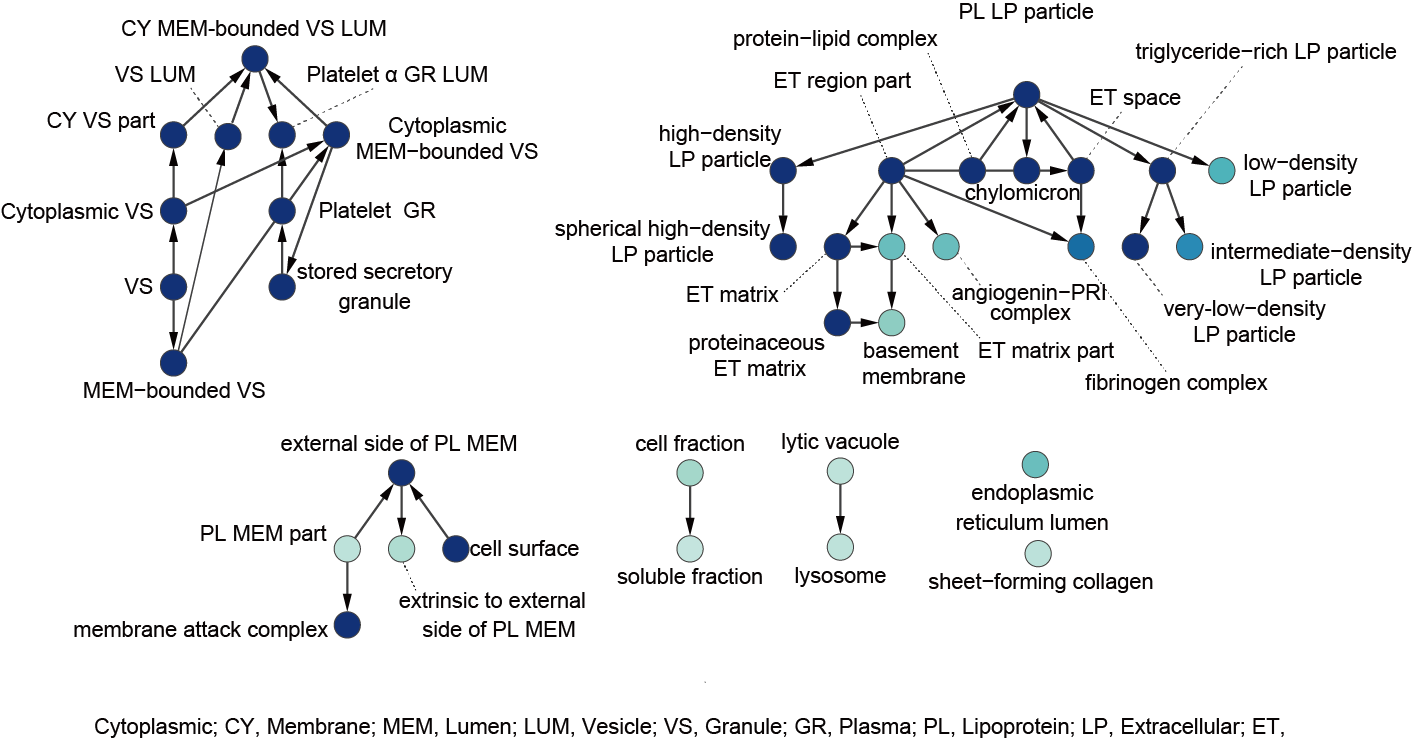
**

**Supplementary figure S1** A GO functional enrichment network of the Pep-Quant library that satisfy a hypergeometric test with FDR correction of P < 0.05. The darker blue color indicates higher enrichment of the proteins to the function. Only the major function or cell components are shown by acronyms and the full GO names are shown in Supplementary Fig X. Acronyms: CY, cytoplasmic; MEM, membrane; Lum, lumen; VS, vesicle; GR, granule; PL, plasma; LP, lipoprotein; ET, extracellular.

**Supplementary figure S2**

**
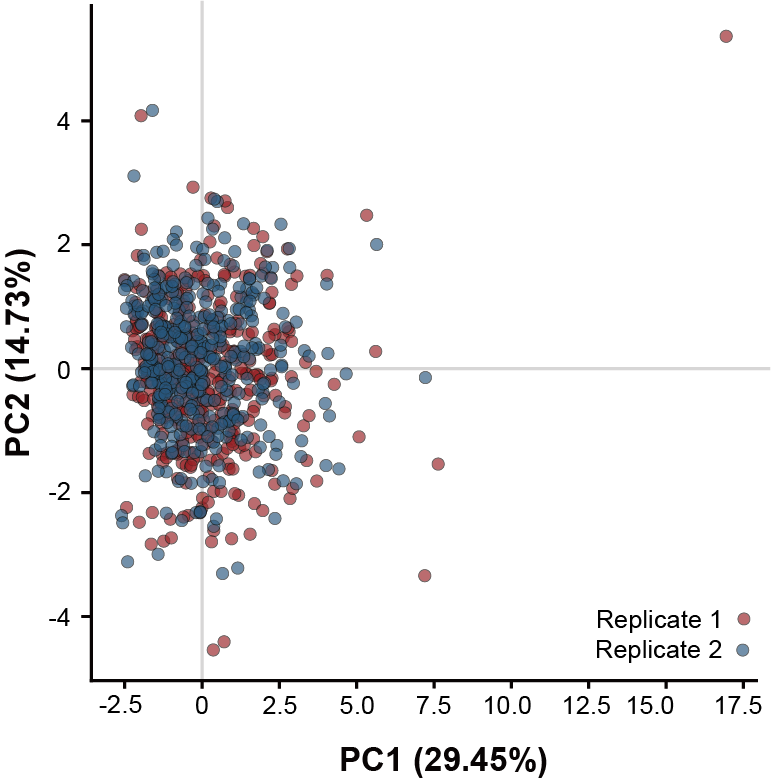
**

**Supplementary figure S2** PCA plot of the replicates.

**Supplementary figure S3**

**
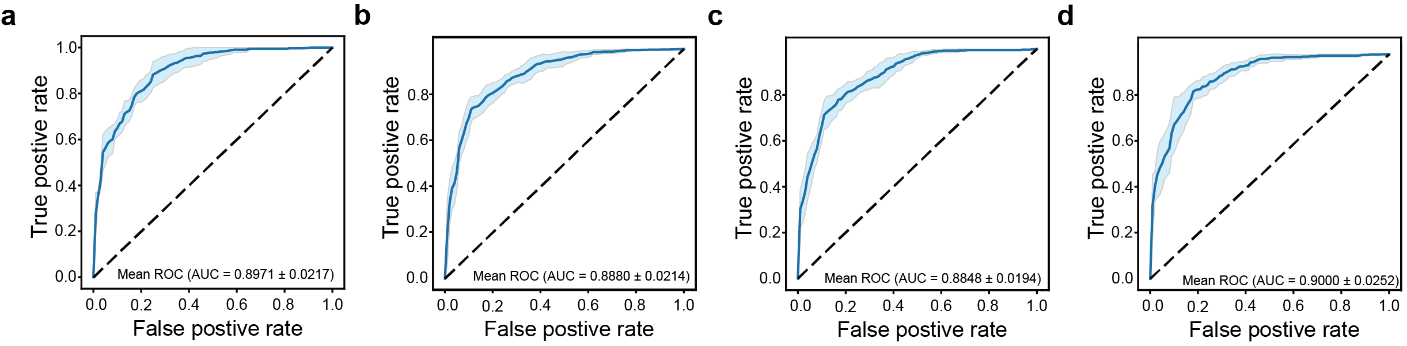
**

**Supplementary Figure S3.** Breast cancer prediction accuracy of different ML models. Area under the curve ROC graph for (A) logistic regression, (B) random forest (C), light GBM and (D) deep learning.

**Supplementary figure S4**

**
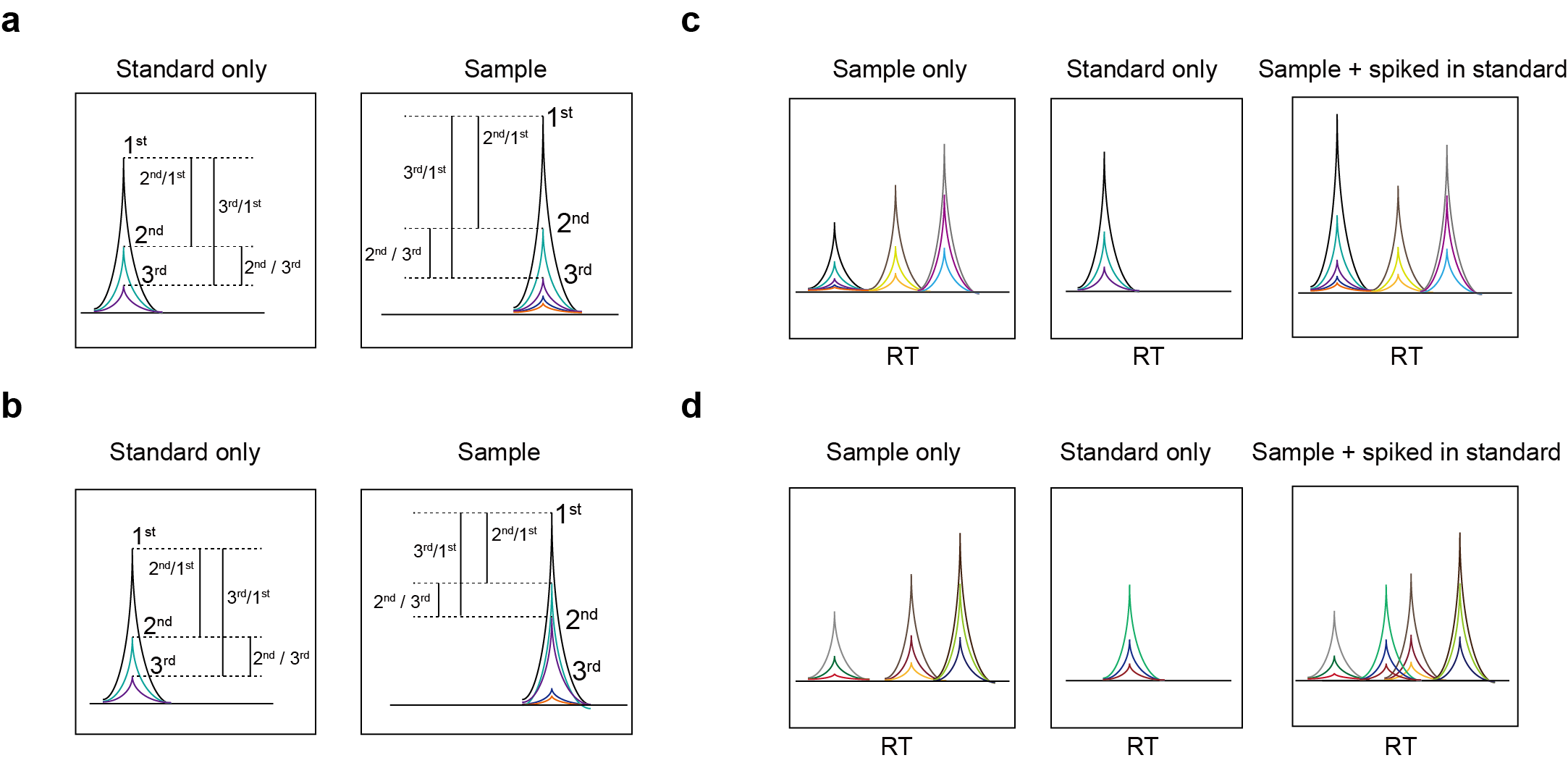
**

**Supplementary figure S4** Schematic diagrams of the peptide identification process. Example of a comparison between the top three peaks of the standards and the samples that are (a) similar and (b) different. Example of a comparison between the standard, sample, and sample with spiked in standards to compare similarity in RT, top three peak ratio to determine a (c) quantifiable peptide and a (d) unquantifiable peptide.

**Supplementary Table S1**

| **Gene** | **Accession** | **Protein name** | **Sequence** |
| --- | --- | --- | --- |
| FN1 | P02751 | Fibronectin | STTPDITGYR |
| FN1 | P02751 | Fibronectin | VDVIPVNLPGEHGQR |
| VWF | P04275 | von Willebrand factor | ILAGPAGDSNVVK |
| VWF | P04275 | von Willebrand factor | VTVFPIGIGDR |
| MMP9 | P14780 | Matrix metalloproteinase 9 | AVIDDAFAR |
| PRG4 | Q92954 | Proteoglycan 4 | AIGPSQTHTIR |
| PRG4 | Q92954 | Proteoglycan 4 | DQYYNIDVPSR |
| THBS1 | P07996 | Thrombospondin 1 | LVPNPDQK |
| APOC1 | P02654 | Apolipoprotein C1 | TPDVSSALDK |
| CHL1 | O00533 | Neural cell adhesion molecule L1 like | VIAVNEVGR |
| B2M | P61769 | Beta-2-microglobulin | IQVYSR |
| LYZ | P61626 | Lysozyme C | STDYGIFQINSR |
| CTSD | P07339 | Cathepsin D | VSTLPAITLK |
| PPBP | P02775 | Platelet basic protein | TTSGIHPK |
| C4BPA | P04003 | C4b-binding protein alpha chain | LSLEIEQLELQR |
| HBD | P02042 | Hemoglobin subunit delta | TAVNALWGK |
| lgals3bp | Q08380 | Galectin-3-binding protein | YSSDYFQAPSDYR |
| MASP1 | P48740 | Mannan-binding lectin serine protease 1 | SDFSNEER |
| APOF | Q13790 | Apolipoprotein F | SGVQQLIQYYQDQK |
| CPB2 | Q96IY4 | Carboxypeptidase B2 | YSFTIELR |
| VCAM1 | P19320 | Vascular cell adhesion protein 1 | SIDGAYTIR |
| GPLD1 | P80108 | Phosphatidylinositol-glycan-specific phospholipase D | GAVYVYFGSK |
| FCGBP | Q9Y6R7 | IgGFc-binding protein | GNPAVSYVR |
| LTF | P02788 | lactotransferrin | YYGYTGAFR |
| FCN2 | Q15485 | Ficolin-2 | VDGSVDFYR |
| PRDX6 | P30041 | Peroxiredoxin-6 | LSILYPATTGR |
| IGF1 | P05019 | Insulin-like growth factor1 | GFYFNKPTGYGSSSR |
| CLU | P10909 | Clusterin | TLLSNLEEAK |
| CHGA | P10645 | Chromogranin-A | ILSILR |
| PIGR | P01833 | Polymeric immunoglobulin receptor | VYTVDLGR |

**Supplementary Table S1** Biomarker candidates for breast cancer after the exploratory quantification with 50 breast cancer and 50 healthy control samples.

**Supplementary Table S2**

| **Gene** | **Protein** | **Sequence** | **Accession No.** |
| --- | --- | --- | --- |
| APOC1 | Apolipoprotein C1 | TPDVSSALDK | P02654 |
| CHL1 | Neural cell adhesion molecule L1 like | VIAVNEVGR | O00533 |
| HBD | Hemoglobin subunit delta | TAVNALWGK | P02042 |
| PIGR | Polymeric immunoglobulin receptor | VYTVDLGR | P01833 |
| MMP9 | Matrix metalloproteinase-9 | AVIDDAFAR | P14780 |
| PRDX6 | Peroxiredoxin-6 | LSILYPATTGR | P30041 |
| THBS1 | Thrombospondin 1 | LVPNPDQK | P07996 |
| FN1 | Fibronectin | STTPDITGYR | P02751 |
| VWF | von Willebrand factor | ILAGPAGDSNVVK | P04275 |
| MASP1 | Mannan-binding lectin serine protease 1 | SDFSNEER | P48740 |
| PRG4 | Proteoglycan 4 | AIGPSQTHTIR | Q92954 |
| CLU | Clusterin | TLLSNLEEAK | P10909 |
| PPBP | Platelet basic protein | TTSGIHPK | P02775 |
| C4BPA | C4b-binding protein alpha chain | LSLEIEQLELQR | P04003 |
| FCGBP | IgGFc-binding protein | GNPAVSYVR | Q9Y6R7 |
| LTF | lactotransferrin | YYGYTGAFR | P02788 |

**Supplementary Table S2** Biomarker candidates for breast cancer after reproducibility check with larger cohort of 96 breast cancer and 95 healthy control samples.

**Supplementary Table S3**

| **Assay characteristic** | **APOC1** | **CHL1** | **CLU** | **PPBP** | **MMP9** | **PRDX6** | **PRG4** | **FN1** | **VWF** |
| --- | --- | --- | --- | --- | --- | --- | --- | --- | --- |
| Lower Limit of  Quantification (ng/uL) | 0.0010 | 0.0008 | 0.0015 | 0.0187 | 0.0014 | 0.0015 | 0.0018 | 0.0026 | 0.0032 |
| Linearity  (Correlation Coefficient) | 0.9996 | 0.9987 | 0.9987 | 0.9996 | 0.9990 | 0.9999 | 0.9997 | 0.9994 | 0.9993 |
| Selectivity  (interference (%)) | 1.6933 | 7.6067 | 0.6483 | 1.7267 | 6.1833 | 0.0000 | 15.6567 | 1.4467 | 0.0000 |
| Accuracy  (%) | 83.3 | 100.0 | 100.0 | 83.3 | 83.3 | 100.0 | 100.0 | 100.0 | 83.3 |
| Precision intra-day  (CV*) | 0.0269 | 0.0361 | 0.0208 | 0.0606 | 0.0280 | 0.0146 | 0.0674 | 0.0289 | 0.0180 |
| Precision inter-day  (CV) | 0.0515 | 0.0482 | 0.0460 | 0.0601 | 0.0622 | 0.0491 | 0.0483 | 0.0487 | 0.0420 |
| Serum Stability in -80 ℃(CV) | 0.0244 | 0.0328 | 0.0247 | 0.0344 | 0.0440 | 0.1115 | 0.0348 | 0.0311 | 0.0460 |
| Analyte Stability in 4 ℃  (CV) | 0.0313 | 0.0425 | 0.0235 | 0.0342 | 0.0624 | 0.1030 | 0.0425 | 0.0357 | 0.0531 |

**Supplementary Table S3** MRM analytical performance evaluation results of nine markers

* CV indicate coefficient of variation

**Supplementary Table S4**

|  | Sample category | Training set (70%) | Test set (30%) | Total (100%) |
| --- | --- | --- | --- | --- |
| Healthy controls | Age Total | **131** | **56** | **187** |
|  | Age 20-39 | 20 | 8 |  |
|  | Age 40-59 | 92 | 36 |  |
|  | Age 60-79 | 19 | 11 |  |
|  | Age 80+ | 0 | 0 |  |
|  | Unknown | 0 | 1 |  |
| Breast cancer | Age Total | **150** | **65** | **215** |
|  | Age 20-39 | 3 | 3 |  |
|  | Age 40-59 | 82 | 35 |  |
|  | Age 60-79 | 51 | 19 |  |
|  | Age 80+ | 4 | 1 |  |
|  | Unknown | 10 | 7 |  |
|  | Stage Total | **150** | **65** | **215** |
|  | Stage 0 | 14 | 2 |  |
|  | Stage 1 | 33 | 22 |  |
|  | Stage 2 | 41 | 16 |  |
|  | Stage 3+ | 10 | 3 |  |
|  | unknown | 52 | 22 |  |
| Other cancers | Other cancers total | **69** | **29** | **98** |
|  | Ovarian | 14 | 6 |  |
|  | Pancreas | 14 | 6 |  |
|  | Thyroid | 14 | 6 |  |
|  | Colon | 14 | 6 |  |
|  | lung | 13 | 5 |  |
|  | Total | 350 | 150 | 500 |

**Supplementary Table S4** Number of samples used for training and testing the machine learning models

**Supplementary Table S5**

| **GO cellular component** | **Genes** |
| --- | --- |
| very-low-density lipoprotein particle | APOC1 |
| triglyceride-rich lipoprotein particle | APOC1 |
| inclusion body | CLU |
| cytoplasmic vesicle part | FN1 PPBP |
| chylomicron | APOC1 |
| spherical high-density lipoprotein particle | CLU |
| fibrinogen complex | FN1 |
| aggresome | CLU |
| Weibel-Palade body | VWF |
| vesicle | VWF, FN1, PPBP, PRDX6 |
| cytoplasmic vesicle | VWF, FN1, PPBP, PRDX6 |
| membrane-bounded vesicle | VWF, FN1, PPBP, PRDX6 |
| cytoplasmic membrane-bounded vesicle | VWF, FN1, PPBP, PRDX6 |
| vesicle lumen | FN1, PPBP |
| cytoplasmic membrane-bounded vesicle lumen | FN1, PPBP |
| platelet alpha granule lumen | FN1, PPBP |
| plasma lipoprotein particle | APOC1, CLU |
| protein-lipid complex | APOC1, CLU |
| stored secretory granule | VWF, FN1, PPBP |
| high-density lipoprotein particle | APOC1, CLU |
| extracellular matrix | VWF, CHL1, FN1, MMP9 |
| extracellular space | APOC1, FN1, PPBP, CLU, MMP9 |
| proteinaceous extracellular matrix | VWF, CHL1, FN1, MMP9 |
| platelet alpha granule | VWF, FN1, PPBP |
| extracellular region | VWF, CHL1, APOC1, FN1, PRG4, PPBP, CLU MMP9 |
| extracellular region part | VWF, CHL1, APOC1, FN1, PPBP, CLU, MMP9 |

**Supplementary Table S5** Cellular components associated with the nine biomarkers for breast cancer.
